# Phylogeny and Molecular Evolution Analysis of PIN-FORMED 1 in Angiosperm

**DOI:** 10.1371/journal.pone.0089289

**Published:** 2014-02-28

**Authors:** Pengkai Wang, Tielong Cheng, Shuang Wu, Fangfang Zhao, Guangping Wang, Liming Yang, Mengzhu Lu, Jinhui Chen, Jisen Shi

**Affiliations:** 1 Key Laboratory of Forest Genetics and Biotechnology, Ministry of Education, Nanjing Forestry University, Nanjing, China; 2 Division of Research Management, Chinese Academy of Forestry, Beijing, China; 3 School of Life Sciences, Huaiyin Normal University, Huaian, Jiangsu, China; 4 State Key Laboratory of Tree Genetics and Breeding, Research Institute of Forestry, Chinese Academy of Forestry, Beijing, China; Beijing Institute of Genomics, Chinese Academy of Sciences, China

## Abstract

PIN-FORMED 1 (PIN1) is an important secondary transporter and determines the direction of intercellular auxin flow. As PIN1 performs the conserved function of auxin transport, it is expected that the sequence and structure of PIN1 is conserved. Therefore, we hypothesized that PIN1 evolve under pervasive purifying selection in the protein-coding sequences in angiosperm. To test this hypothesis, we performed detailed evolutionary analyses of 67 PIN1 sequences from 35 angiosperm species. We found that the PIN1 sequences are highly conserved within their transmembrane regions, part of their hydrophilic regions. We also found that there are two or more *PIN1* copies in some of these angiosperm species. PIN1 sequences from Poaceae and Brassicaceae are representative of the modern clade. We identified 12 highly conserved motifs and a significant number of family-specific sites within these motifs. One family-specific site within Motif 11 shows a different residue between monocots and dicots, and is functionally critical for the polarity of PIN1. Likewise, the function of PIN1 appears to be different between monocots and dicots since the phenotype associated with PIN1 overexpression is opposite between Arabidopsis and rice. The evolution of angiosperm *PIN1* protein-coding sequences appears to have been primarily driven by purifying selection, but traces of positive selection associated with sequences from certain families also seem to be present. We verified this observation by calculating the numbers of non-synonymous and synonymous changes on each branch of a phylogenetic tree. Our results indicate that the evolution of angiosperm PIN1 sequences involve strong purifying selection. In addition, our results suggest that the conserved sequences of PIN1 derive from a combination of the family-specific site variations and conserved motifs during their unique evolutionary processes, which is critical for the functional integrity and stability of these auxin transporters, especially in new species. Finally, functional difference of PIN1 is likely to be present in angiosperm because the positive selection is occurred in one branch of Poaceae.

## Introduction

The plant hormone auxin is involved in many aspects of plant growth and development, including embryogenesis, organogenesis, tissue differentiation and gravitropism[1], [2]. At the same time, auxin is required for the division, enlargement and differentiation of individual plant cells. Auxin as signal molecule between cells, tissues and organs contributes to the coordination and integration of growth and development in the whole plant and to physiological responses of plants to environmental signal[3], [4]. There is evidence that auxin plays a central role in the majority of plant hormonal functions, as various hormones interact with auxin[5]. Indole-3-acetic acid (IAA) is considered as the primary naturally occurring auxin in plants[6]. Recently, some experimental evidence demonstrates the positive feedback loop consisting of auxin and its efflux carrier PIN-FORMED1 (PIN1) plays an important role in the spatiotemporal regulation of organ formation[7]. For PIN1 transport auxin, they regulate a number of developmental processes, including morphogenesis, organogenesis, and stress responses[5], [8], [9]. They are oriented in the plasma membrane such that they mediate the directional flux of auxin within tissues and generate auxin gradients that influence development[10], [11].

A number of studies have shown that some amino acids and motifs in AtPIN1 determine the location and function. PIN1 polarity is controlled by the antagonistic actions of the protein kinase, PINOID, and protein phosphatase 2A[12], [13]. In *Arabidopsis*, PIN polarity, and therefore the distribution of auxin, depends on the phosphorylation of the conserved residues Ser337 and Thr340[13]. Two motifs are particularly important for the intracellular trafficking of auxin by PIN1. The first, a TPRXS(N/S) motif, is located within the amino-terminal portion of the hydrophilic loop and is found in three copies[14]. This motif is important for the trafficking of PIN1 from the endoplasmic reticulum to the plasma membrane[15]. The second is a tyrosine-based internalization motif that is important for recruitment of proteins into clathrin-dependent vesicles[16]. These conclusion were made by the results of Arabidopsis experiments and there were no reports in other plant. Therefore, Bioinformatic analysis of specific amino acids and motifs in other plant PIN1 might offer clues to the *PIN1* orthologs functional research.

The structure of intron/exon and coding sequences in *PIN1* orthologs of other angiosperm plants is highly conserved. Bioinformatic methodologies have predicted that each hydrophobic region contains four/five transmembrane helices and that structural similarities exist between PIN1 and other membrane-bound secondary transport proteins that use the trans-membrane electrochemical gradient as an energy source for transport[8]. In previous studies, there were some reviews about PIN1 family in green plant but less evolutional analysis[16], [17]. The literature has not yet described the PIN1 evolutional history in angiosperm. PIN1 protein structure and more detailed characterization of the function are important topics for further studies.

For this report, we examined the evolution of 67 angiosperm PIN1 sequences from 38 plant species by conducting phylogenetic analyses, followed by analyses of specific PIN1 domains and motifs. Analysis of the types of evolutionary pressures that affected the sequences yielded the expected results with the sequences having, in the main, experienced strong purifying selection (rather than pervasive positive selection) throughout angiosperm evolution. However, analyses also showed that some sites within the sequences had been under positive selection, despite little evidence for positive pressure influences generally on these genes. In particular, positive selection on such *PIN1* protein-coding sites is apparent for during formation of Brassicaceae and within Poaceae. By analyzing the evolution of *PIN1*, rules concerning the evolution of highly conserved genes (in terms of function and sequence) may be revealed.

## Methods

### Sequence data

We retrieved the *A. thaliana* PIN1 protein and protein-coding sequence from the Arabidopsis Information Resource database (www.arabidopsis.org). A BLASTP search was then performed using the AtPIN1 sequence as the query to retrieve PIN1 sequences from the NCBI (www.ncbi.nlm.nih.gov) and Phytozome databases[18] (www.phytozome.org). The identified sequences were from the plant species *Brachypodium distachyon*, *Hordeum vulgare*, *Oryza sativa*, *Panicum virgatum*, *Sorghum bicolor*, *Setaria italica*, *Triticum aestivum*, *Zea mays*, *Arabidopsis lyrata*, *Arabidopsis thaliana*, *Brassica rapa*, *Capsella bursa-pastoris*, *Cardamine hirsuta*, *Capsella rubella*, *Thellungiella halophila*, *Cicer arietinum*, *Glycine max*, *Lupinus albus*, *Medicago truncatula*, *Pisum sativum*, *Phaseolus vulgaris*, *Fragaria vesca*, *M. domestica*, *Prunus persica*, *Cucumis sativus*, *Momordica charantia*, *Gossypium raimondii*, *Theobroma cacao*, *Manihot esculenta*, *Populus trichocarpa*, *Citrus clementina*, *Citrus sinensis*, *Nicotiana tabacum*, *Solanum lycopersicum*, *Solanum tuberosum*, *Vitis vinifera*, *Carica papaya*, and *Amborella trichopoda* (Table S1). All selected *PIN1* protein sequences contain one hydrophobic domain and two hydrophilic domains. In the prephylogenetic analysis of these *PIN1* protein sequences with *Arabidopsis thaliana* PIN family, make sure all *PIN1* genes as well as *AtPIN1* cluster together to form a large group. Some genes outside the PIN1 group will not be analyzed in this report. The NCBI annotations for these sequences were used to delineate the hydrophobic and hydrophilic domains, which were then analyzed separately.

### Multiple-sequence alignment and phylogenetic-tree construction

**Figure 1:**
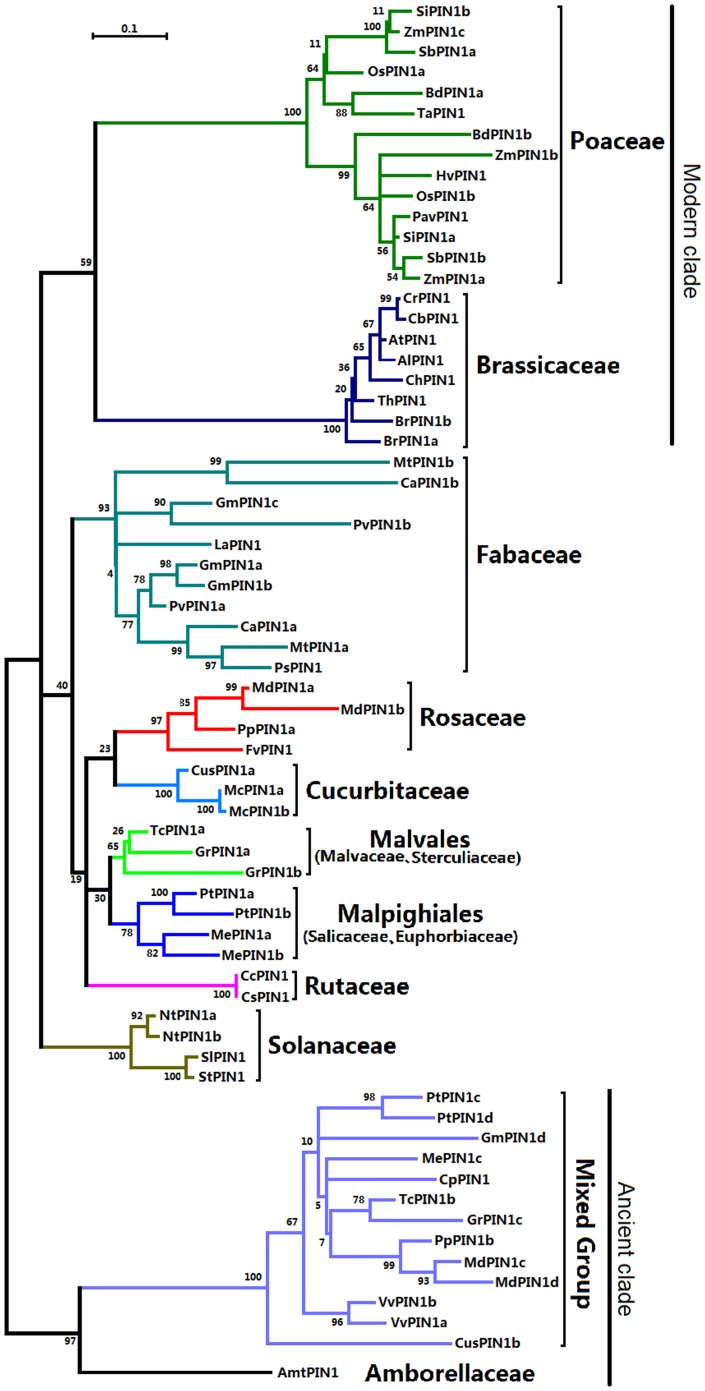
Maximum-likelihood phylogenetic tree of the angiosperm PIN1. The ML tree was constructed based on the protein sequences of angiosperm PIN1 using MEGA5.2 with 1000 bootstrap replications and Jones-Taylor-Thornton (JTT) + Gamma Distributed model (Discrete Gamma Categories = 5). These PIN1 protein sequences were searched from Poaceae, Brassicaceae, Fabaceae, Rosaceae, Cucurbitaceae, Malvales, Malpighiales, Rutaceae, Solanaceae, Vitaceae, Caricaceae and Amborellaceae. The scale bar indicates the branch length that corresponds to 0.1 substitutions per site. The species and accession numbers are listed in Table S1.The abbreviations used are as follows: *Bd*, *Brachypodium distachyon*; *Hv*, *Hordeum vulgare*; *Os*, *Oryza sativa*; *Pav*, *Panicum virgatum*; *Sb*, *Sorghum bicolor*; *Si*, *Setaria italica*; *Ta*, *Triticum aestivum*; *Zm*, *Zea mays*; *Al*, *Arabidopsis lyrata*; *At*, *Arabidopsis thaliana*; *Br*, *Brassica rapa*; *Cb*, *Capsella bursa-pastoris*; *Ch*, *Cardamine hirsuta*; *Cr*, *Capsella rubella*; *Th*, *Thellungiella halophila*; *Ca*, *Cicer arietinum*; *Gm*, *Glycine max*; *La*, *Lupinus albus*; *Mt*, *Medicago truncatula*; *Ps*, *Pisum sativum*; *Pv*, *Phaseolus vulgaris*; *Fv*, *Fragaria vesca*; *Md*, *Malus domestica*; *Pp*, *Prunus persica*; *Cus*, *Cucumis sativus*; *Mc*, *Momordica charantia*; *Gr*, *Gossypium raimondii*; *Tc*, *Theobroma cacao*; *Me*, *Manihot esculenta*; *Pt*, *Populus trichocarpa*; *Cc*, *Citrus clementina*; *Cs*, *Citrus sinensis*; *Nt*, *Nicotiana tabacum*; *Sl*, *Solanum lycopersicum*; *So*, *Solanum tuberosum*; *Vv*, *Vitis vinifera*; *Cp*, *Carica papaya*; *Amt*, *Amborella trichopoda*.

**Figure 2:**
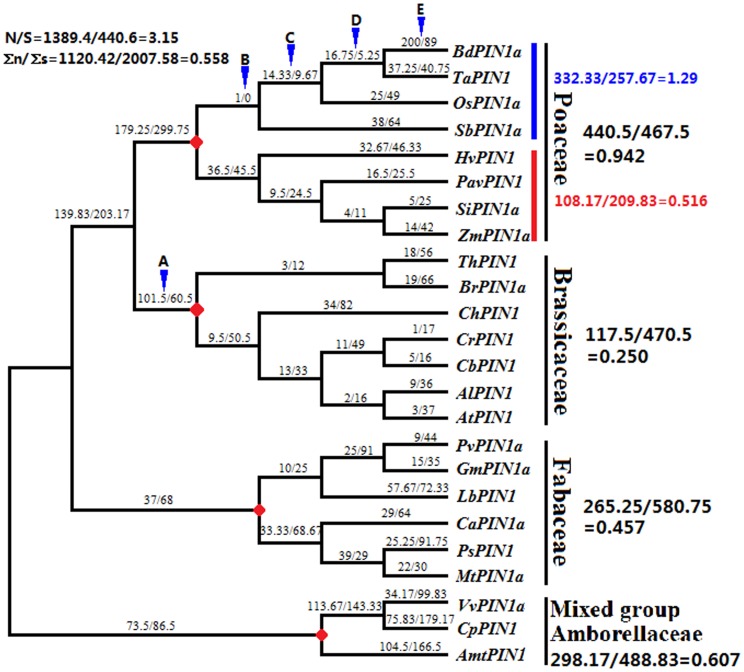
Numbers of non-synonymous (n) and synonymous (s) substitutions in four groups. A phylogenetic tree was constructed using 24 *PIN1* protein-coding sequences. Shown above each branch is the n/s value. The n/s values for the groups formed by Poaceae, Brassicaceae, Fabaceae, and the mixed group including *AmtPIN1* (and excluding their ancestral branches) are shown below their names. The three solid, red nodes represent the positions of the ancestors of the four groups. N and S are the calculated number of non-synonymous and synonymous sites, respectively. Blue arrows (A–E) indicate branches that have undergone positive selection.

Alignment of the sequences was performed by ClustalX v2.0[19] and followed by manual corrections. Sequence relationships were inferred using the maximum-likelihood method. Maximum-likelihood phylogenies were constructed using MEGA 5.2[20]. In the phylogenetic-trees of Figure 1 and S1, the bootstrap value derived from 1,000 replicates was assumed to represent the evolutionary history of the included taxa. Before tree construction analysis, MEGA 5 had been used to determine that the best model for maximum-likelihood analysis of the sequences and found to be the Jones-Taylor-Thorton+ Gamma model. The phylogenetic-tree of Figure 2 was constructed by the Neighbor-joining method within Poisson model using 24 typical *PIN1* protein-coding sequences from the modern clade, Fabaceae and the ancient clade. Sequences were assigned to different subfamilies on the basis of their similarities and groupings within the phylogenetic tree.

### Identification of sequence motifs

To identify motifs shared among related proteins within the PIN1, the MEME[21] motif search tool was used with its default settings. The maximum number of possible motifs was set to 20, and the maximum width was 300. Identified motifs were annotated using SMART (http://smart.embl-heidelberg.de/)[22]and Pfam(http://pfam.sanger.ac.uk/)[23].

### Selective pressure analysis of PIN1 sequences

**Figure 3:**
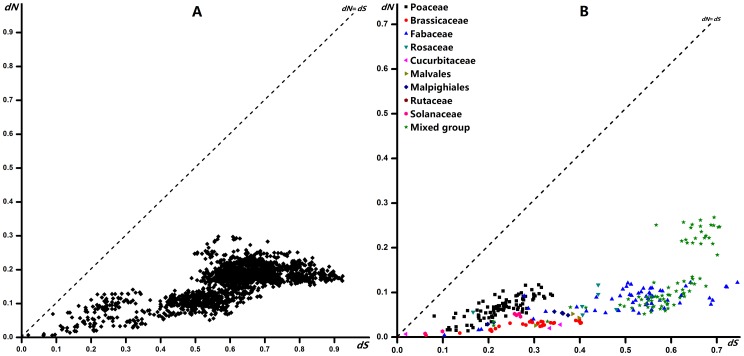
Pairwise comparison plots of dN and dS values for all angiosperm PIN1 genes (A) and each family (order) PIN1 genes (B).

The numbers of non-synonymous substitutions per nonsynonymous site (*dN*) and that of synonymous nucleotide substitutions per synonymous site (*dS*) was determined using the KaKs_Calculator[24] adjustments made for the transition/transversion ratio (Figure 3). Differences between *dN* and *dS* values were analyzed using Z-test in MEGA 5.2[20], with standard errors derived from 1000 bootstrap replicates. Ancestral PIN1 sequences at all interior nodes of the three family trees were inferred on the basis of the phylogeny of 18 angiosperm species using ANC-GENE software [25], [26]. The number of s and n substitutions were then calculated for each branch of the tree.

The site (M7 and M8) and branch models of the maximum-likelihood method were used to test for positive selection at individual sites within a specific lineage and at different sites, respectively. These analyses were performed using codeML implemented in PAML 4.2[27]. Site model was used in PIN1 genes by comparing the selection model M8 with the null model M7. Suppose that the ω (the nonsynonymous to synonymous substitution rate ratio, also known as *dN*/*dS*) values is a beta distribution between 0 and 1 in M7 and there are no sites under positive selection. M8 is similar with M7 except that there is another type of sites (ω>1). The best fit model was found by the Likelihood ratio tests (LRT) of different models for the data. Statistical significance was showed by comparing twice the log likelihood difference between models to a χ^2^ statistic with the degrees of freedom equal to the difference in number of parameters between models. The branch models allow the ω ratio to vary among branches in the phylogeny and are useful for detecting positive selection acting on particular lineages. A significant difference in the ω rate ratio between different branches was calculated by comparing a free-ratio model (model = 1), which assumes an independent ω ratio for each branch, with a model given an average ratio to all lineages (model = 0).

## Results and Discussion

### Numerical expansion of PIN1 within some families of angiosperm

To investigate the evolution of PIN1 sequences, 67 amino acid sequence data for PIN1 was obtained from 38 species that belong to 12 angiosperm families. The member of PIN1 isoforms in each of these species is listed in Table S1. Most of these angiosperm species possess two *PIN1* copies, with the exception of *Z. mays*, *Glycine max*, *M. domestica*, *G. raimondii*, and *P. trichocarpa*, which have three or four *PIN1* copies. Similar results have been reported elsewhere[28], [29]. Only one *PIN1* was found for *H. vulgare*, *P. virgatum*, *T. aestivum*, *A. thaliana*, *C. bursa-pastoris*, *C. hirsuta*, *C. rubella*, *T. halophila*, *L. albus*, *P. sativum*, *F. vesca*, *C. clementina*, *C. sinensis*, *S. lycopersicum*, *S. tuberosum*, *C. papaya*, *A. trichopoda*. The absence of a second *PIN1* in these species may be a consequence of incomplete or low-quality sequence data (except for *A. thaliana*, *C. bursa-pastoris*, *C. hirsuta*, C. *rubella*, *T. halophila*).

With the exception of *B. rapa*, all species from Brassicaceae, only a single *PIN1* sequence was found. Four copies of PIN1 sequences from maize (*ZmPIN1a-d*) and nine *PIN1* sequences from poplar were identified by a comprehensive Blast search of public databases using the AtPIN1 sequence as the query. Sequence alignment revealed that, the short stretches of overlapping sequences within ZmPIN1a–d sequences were presented. As *ZmPIN1d* is especially expressed in maize[28], only the ZmPIN1a–c sequences were used for phylogenetic tree building. In addition, some of the nine poplar sequences are incomplete, suggesting that they might be from pseudogenes. Therefore, only four poplar sequences PtPIN1a-d were chosen for tree building. Because multiple sequences were identified for most of the species, the results suggest that PIN1 must have expanded during angiosperm evolution.

### Phylogenetic analysis of PIN1 protein sequences

All reported and predicted angiosperm PIN1 sequences (67 in all) were retrieved from the plant genome (Phytozome and NCBI) and protein databases (NCBI) and used to construct a maximum-likelihood phylogenetic tree (Figure 1). Most of the sequences from angiosperm species within a single family clustered together to form an independent group. The Poaceae, Brassicaceae, Fabaceae, Rosaceae, Cucurbitaceae, Rutaceae, and Solanaceae families are well clustered in Figure 1 (bootstrap value>93%). Bootstrap values for the Malvales and Malpighiales orders are smaller (65% and 78%, respectively) because sequences from different families were included. Bootstrap values associated with all higher clades are generally relatively smaller (bootstrap values<60%). Intriguingly, PIN1 sequences from *V. vinifera* (VvPINIa and b) and Carica papaya (CpPIN1) are found together in a statistically supported branch (bootstrap value = 100%). We denoted these sequences the mixed-group clade because it also contains sequences from Fabaceae, Rosaceae, Cucurbitaceae, Malvales, and Malpighiales. *A. trichopoda* PIN1 (AmtPIN1) is part of an independent branch within the phylogenetic tree that is is grouped with the mixed group to form a clade (bootstrap value = 97%). These results indicate that most of these sequences are specific at the family level.

In the phylogenetic tree, genes from other families and orders cluster together to form a larger group (except for the mixed group and AmtPIN1) (Figure 1). Within this large group, the sequences cluster into three independent clades (with small bootstrap values except for those of Solanaceae) indicating that they may have arisen from a common ancestor. Within these three clades, the sequences from the same family clustered together to form subgroups. Sequences from the clade containing the mixed group and that of AmtPIN1 may represent the ancient PIN1 clade because *A. trichopoda* represents the most ancestral angiosperm[30]. Interestingly, the ancient group contains sequences from Fabaceae, Rosaceae, Cucurbitaceae, Malvales, Malpighiales, *V. vinifera* and *C. papaya*, but not those from Poaceae, Brassicaceae, and Solanaceae, which would be suggested that, the PIN1 of angiosperm may be derived from a common ancestor, and that evidence of the evolutionary processes may be preserved in some of these species. This evidence may have been lost in Poaceae and Brassicaceae, indicating that Poaceae and Brassicaceae *PIN1* are relatively modern genes. Additional sequence data is needed to prove that PIN1 of Rutaceae and Solanaceae belongs to a modern clade. This phylogenetic analysis indicates that duplication of *PIN1* occurred during the evolution, especially in Poaceae and Fabaceae. This gene-duplication event has been confirmed in maize[28]. Even though, there was a significant difference between the PIN1 evolutionary tree and APG Systems [31], [32]. *PIN1* molecular evolutionary process in angiosperm only can be reflected by the PIN1 evolutionary tree (Figure 1) containing 67 PIN1 angiosperm genes but can not stand for the true evolutionary relationship of families in angiosperm.

### Strong purifying selection affected the evolution of angiosperm PIN1

Different types of selective pressure can be revealed by the rate ratio (*dN/dS*) of non-synonymous (N) to synonymous (S) genetic changes. The values of *dN/dS* ratios<1, 1, and >1 were the indicator for purifying selection, neutral evolution, and positive selection, respectively[33], [34]. To study selective pressures associated with angiosperm *PIN1*, the values for *dN* and *dS* distances were calculated for the 67 *PIN1* genes from the 38 species (7 families and 2 orders in all). Pairwise comparisons of *dN* and *dS* values within all sequences, and within those of each family (order) were performed by MEGA 5.2 using the modified Nei-Gojobori method. For 2211 pairwise comparisons involving these sequences, significantly fewer non-synonymous than synonymous changes were found (*dN* << *dS*, *p*<0.01, *Z-test*, Figure 3A). Points for all sequences were found near the dS axis and away from the diagonal, indicating that *dN* = *dS* (Figure 3A) and strongly suggesting that purifying selection dominated the selection process during the evolution of angiosperm PIN1. Similar *dN/dS* values were obtained for each family (order) that contained the mixed group (*p*<0.01, *Z-test*, Figure 3B), indicating that purifying selection acted on PIN1 within each family (order) of angiosperm.

Average *dN* and *dS* values for the sequences from each family (order) were calculated, revealing in each case a dN/dS value that was significantly <1 (p<0.01, Z-test), i.e., average *dN* values were significantly smaller than were *dS* values (Figure S1). To prove that purifying selection drove the evolution of the PIN1 protein-coding sequences, we calculated average *dN* and *dS* values for the sequences within the phylogenetic tree (Figure 1). The average *dN* and *dS* values are 0.164 and 0.612, respectively, and the average *dN/dS* value is 0.268, supporting that angiosperm PIN1 sequences were subjected to purifying selection during evolution.

Within each family (order) however, alignment of the corresponding sequences revealed very little variation. To examine if individual amino acid sites within the sequences are under positive selection, we calculated ω rate ratios within the sequences of the families (order) using the free-ratio model in PAML 4.2[27]. When sites within the Malvales and Solanaceae sequences were analyzed, the codon-substitution free-ratio model (M1), which allows for different ω values among the branches, did not fit the data any better than did one-ratio model (M0), which assumes a homogeneous mean ω value for all lineages (Table S2). The values of ω for these PIN1 genes (0.024–0.159) are substantially <1. For the sequences from Poaceae, Brassicaceae, Fabaceae, Rosaceae, Cucurbitaceae, Malpighiales, and the mixed group, the M1 model fit the data better than M0 model, suggesting that sequences from different families experienced different selective pressures. Therefore, the site-model (M7 and M8) was used to examine whether the positive selection drove *PIN1* evolution within each family (order). No significant evidence for positive selection was detected for the sequences from any family (order) (ω<1, Table 1), supporting the conclusion that purifying selection drove the evolution of angiosperm *PIN1* protein-coding sequences.

**Table 1:** Site model (M7 vs. M8) test for each family PIN1 genes.

Family	*dN*/*dS*	Estimates of parameters		InL		2Δl	P
/Order	(M7)	M7	M8	M7	M8		-value
Poaceae*	0.0449	p = 0.16 q = 3.08	p0 = 0.99(p1 = 0.01) p = 0.18 q = 4.31 ω = 1.00	−6573.41	−6569.48	7.87	0.0050
Brassicaceae	0.0686	p = 0.07 q = 0.94	p0 = 1.00(p1 = 0.00) p = 0.07 q = 0.94 ω = 1.00	−5388.78	−5388.78	0.00	0.9814
Fabaceae	0.0955	p = 0.18 q = 1.62	p0 = 0.99(p1 = 0.00) p = 0.20 q = 2.15 ω = 1.12	−7105.42	−7104.10	2.64	0.1044
Rosaceae	0.1056	p = 0.12 q = 0.98	p0 = 1.00(p1 = 0.00) p = 0.12 q = 0.98 ω = 1.00	−4645.86	−4645.86	0.00	0.9925
Cucurbitaceae	0.0432	p = 0.43 q = 9.02	p0 = 1.00(p1 = 0.00) p = 4.00 q = 99.00 ω = 2.54	−2964.52	−2964.03	0.96	0.3265
Malvales	0.0877	p = 0.29 q = 2.89	p0 = 0.99(p1 = 0.01) p = 0.38 q = 4.17 ω = 1.00	−3725.85	−3725.83	0.03	0.8585
Malpighiales	0.0964	p = 0.13 q = 1.16	p0 = 0.94(p1 = 0.06) p = 4.37 q = 99.00 ω = 1.00	−4205.49	−4204.44	2.09	0.1484
Solanaceae	0.1105	p = 0.01 q = 0.07	p0 = 0.88(p1 = 0.12) p = 0.01 q = 0.45 ω = 1.00	−3256.82	−3256.70	0.24	0.6235
Mixed group	0.0548	p = 0.21 q = 3.31	p0 = 1.00(p1 = 0.00) p = 0.21 q = 3.56 ω = 3.40	−8962.26	−8961.87	0.78	0.3771

lnL: the log-likelihood difference between the two models; 2Δl: twice the log-likelihood difference between the two models.

*: In the analysis of Poaceae, the P-value is less than the significance level 0.05, indicating that the M8 model fitted the data better than M7 model. However, the estimate of ω in M8 was less than (99% sites) or equal to 1 (1% sites), indicating no positive selection.

### Highly conserved motifs and family-specific sites within angiosperm PIN1

**Figure 4:**
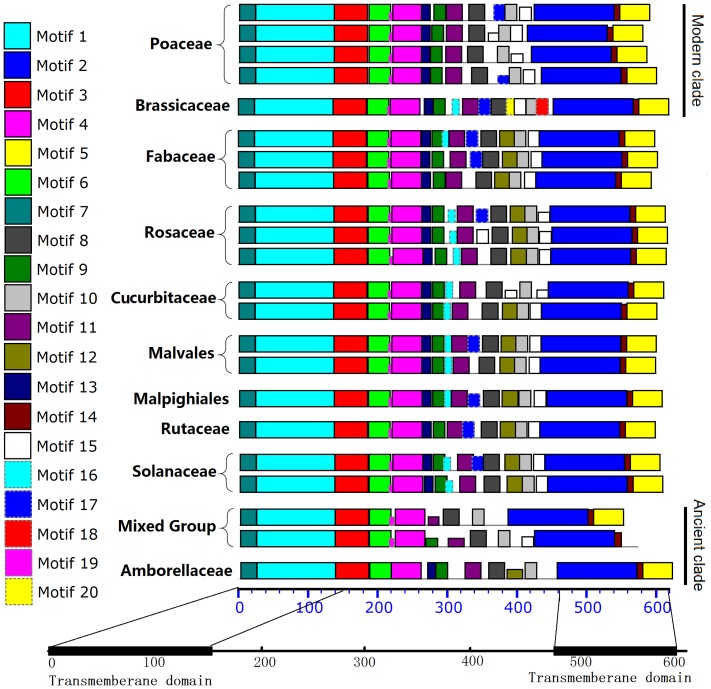
Motif distributions of the angiosperm PIN1 sequences. A schematic representation of motifs obtained using MEME within the sequences is displayed. Different motifs are highlighted by different colored boxes, Details concerning individual motifs are given in Figure S3.

**Figure 5:**
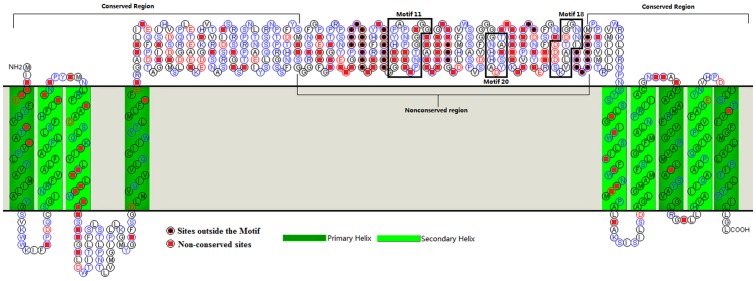
A model of AtPIN1 secondary structure. A predicted membrane-spanning PIN1 structure was generated using the topology-prediction program SOSUI (http://bp.nuap.nagoya-u.ac.jp). Motifs 18 and 20 are specific to Brassicaceae. The distribution of non-conserved sites and the conserved and non-conserved regions are marked in the model.

For both of the amino acid sequence alignment and *dN/dS* values indicated a limited amino acid sequence variation among the sequences. The distribution of motifs was investigated within the angiosperm sequences. The AtPIN1 sequence was used as the query to identify one typical PIN1 sequence in each family. This approach yielded 38 sequences. A maximum-likelihood phylogenetic tree for these sequences was then built (Figure S2) and the result was similar to Figure 1. Motif analysis of the 38 sequences was performed using MEME/MAST[21]. The results (Figure S3) revealed that the sequences contain 12 highly conserved motifs (Motifs 1–11, 14, existing in all typical PIN1). Motifs 1–8 and 14 are found in conserved sequence regions including the two transmembrane regions and the first third of the hydrophilic domain. Motifs 9–11, however, are located within non-conserved regions (Figure 4, 5). Comparisons of motif distributions revealed differences among the sequences when different clades or families were compared (Figure 4). In the modern-clade sequences (Poaceae and Brassicaceae), Motif 12 is absent and the sequences of Motifs 18 and 20 are characteristic only for the Brassicaceae sequences. In addition, Motif 16 is not found in the Poaceae sequences. Within non-conserved regions of the ancient-clade PIN1 sequences, motif depletion is very common. Motifs 5 (VvPINa), 9 (VvPINa), 12 (VvPIN1a, CpPIN1, and AmtPIN1), 13 (VvPIN1 and CpPIN1), 15 (VvPINa and AmtPIN1), 17 (VvPIN1a, CpPIN1, and AmtPIN1), and 19 (AmtPIN1) are missing. Nearly complete conservation of a PIN1 motif implies that it is functionally important and that its formation and distribution among PIN1 sequences from different species was a significant evolutionary event. The combination of family-specific sequence variations and well-conserved motifs may have helped maintain the function of PIN1 as new species were formed. This should suggest that the Brassicaceae PIN1 is more evolutionary than Poaceae.

**Figure 6:**
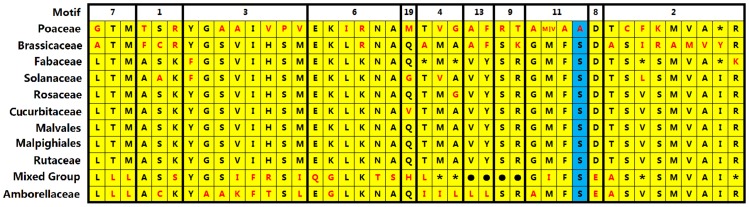
Family-specific sites. “*” means the amino acid is diversified in this position. “•”means there is no amino acid in this position.

Non-conserved sites within PIN1 account for only ∼17% of the total protein sequence (Figure S4). Half of these non-conserved sites are located in a non-conserved region (Figure 5). AtPIN1 polarity is associated with a Motif-11 residue in the non-conserved region[13], which is Ser in all dicotyledon PIN1 sequences, but Ala in Poaceae sequences (Figure 6). Motif 11 is highly conserved, indicating that it serves an important function in angiosperm PIN1. On the basis of this analysis some non-conserved sites are isolated from conserved motifs at the family (order) level (Figure 6). Most of these specific sites are found in the modern and ancient clade sequences, whereas there are no specific sites in Rutaceae, Malvales, and Malpighiales sequences. To date, there is no evidence that these specific sites serve a function (except the Ser/Ala site in Motif 11), but our observations suggest that they should be an important focus of future research concerning PIN1.

### Remnants of positive selection associated with the formation of new families

Our motif and family-specific site analyses verified high levels of conservation at the family level. To examine evolutionary pressures between families (orders), ancestral coding sequences for each family (order) were inferred using ANC-GENE[25], [26]. Posterior probabilities associated with these inferences indicated excellent reliability because the average accuracy is >85%. We calculated ω value for various residue sites identified as ancestral sites among the various families (order) using the M0 and M1 model in PAML 4.2[27]. The M7 and M8 models were then used to examine if positive selection had driven evolution among angiosperm families (order) (Table 2). The free-ratio model fit the data better than did the one-ratio model (*p*<0.05), suggesting that the ancestral sequences experienced variable selective pressures. Significant evidence for positive selection was detected in the ancestral sequences (ω>1) (Table 2), indicating that positive selection drove the evolution of the ancestral PIN1 protein-coding sequences. The M7 and M8 model analysis accounted for selective pressure variation among ancestral sequences and detected positive selection at individual sites[35], [36]. This model exhibits a good fit to the data (*p* = 0.043, χ2-test), and shows that positive selection drove the formation of *PIN1* of new family. In addition, we detected positive selection associated with three sites with posterior probabilities of 91.4%, 92.3%, and 95.1%.

**Table 2:** Branch and site models test for ancestral protein sequences of each families (orders) PIN1 genes.

Model	dN/dS	Estimates of parameters	InL	2Δl	P value
one-ratio	0.0745	–	−10734.861	100.735	0.0000
free-ratio	(one-ratio)	–	−10684.493		
M7	0.1012	p = 0.15 q = 1.26	−10284.168	8.151	0.0043
M8	(M7)	p0 = 0.98(p1 = 0.02) p = 0.17 q = 2.04 ω** = 1.11642**	−10280.093		

Positively selected sites: 308G(0.914), 311P(0.951), 315G(0.923).

Analyses of selective pressures, motifs, family-specific sites, and ancestral sequences all indicate that the primary force associated with angiosperm PIN1 protein-coding sequence evolution is purifying selection, particularly during the formation of new species within a family. When a new family formed, conserved protein motifs arose within non-conserved regions and traces of positive selection focused on conserved regions of the gene. This evolutionary pattern ensured that PIN1 function was maintained as *PIN1* evolved.

### Positive selection within the modern clade of angiosperm *PIN1* protein-coding sequences

Although purifying selection appears to be the main selective pressure during the evolution of angiosperm *PIN1* protein-coding sequences, we found some evidence for positive selection. To statistically test for positive selection in these sequences, the numbers of non-synonymous (n) and synonymous (s) substitutions associated with each branch of phylogenic tree containing 24 typical *PIN1* protein-coding sequences from the modern clade (Poaceae and Brassicaceae), Fabaceae, and the ancient clade (*VvPIN1a*, *CpPIN1*, and *AmtPIN1*) using MEGA 5.2 (Neighbor-joining method and Poisson model) (Figure 2) were calculated. These results were compared with the number of N and S sites[37]. Similar to the phylogenetic tree in Figure 1, four groups, Poaceae, Brassicaceae, Fabaceae, and the mixed group, are well classified in this maximum-likelihood tree (bootstrap>90%). Ancestral *PIN1* protein-coding sequences were inferred at all interior nodes of the tree using ANC-GENE[25], [26]. Posterior probabilities for these inferences are reliable because the average accuracy is >85%. The numbers of n and s substitutions on each branch of the maximum-likelihood tree were calculated using the KaKs_Calculator with the Nei–Gojobori method [24] (Figure 2). The number of n and s substitutions for all branches is 1120.42 and 2007.58, respectively. The number of N and S sites is 1389.4 and 440.6, respectively. As such, the n/s and N/S values are 0.558 and 3.15, respectively. This represents a statistically significant difference between the n/s and N/S values (n/s << N/S, *p*<0.01, Fisher's Exact Test). These calculations indicate purifying selection during the entire history of *PIN1* protein-coding sequence evolution, which is consistent with our pairwise comparison of the data (Figure 3, S1).

The n/s values are 440.5/467.5 = 0.942 for Poaceae, 117.5/470.5 = 0.250 for Brassicaceae, and 265.25/580.75 = 0.475 for Fabaceae. These values are each significantly smaller than the N/S value of 3.15 (*p*<0.01, Fisher's exact test), which suggests strong purifying selection on *PIN1* protein-coding sequences in these three lineages. These results are well supported by the results shown in Figures 3 and 4. Although the major selective pressure appears to be purifying selection in these three lineages, the effects of positive selection are obvious in some branches. During formation of the Brassicaceae branch there was, apparently, weak positive selection (arrow in Figure 2, arrow A, n = 101.5, s = 60.5), but positive selection did not affect Brassicaceae after its formation. Interestingly, the exact opposite event happened in Poaceae. Positive selection is evident within the family (arrows in Figure 2, arrow B–E), but positive selection apparently did not occur during its formation. In addition, Poaceae species could be divided into two linkage groups, one undergoing positive selection and the other undergoing purifying selection. The n/s value for the positive-selection linkage is 1.29 (332.33/257.67), which is significantly larger than the n/s value for the purifying-selection linkage 0.516 (108.17/209.83, *p*<0.01, Fisher's exact test). Given the numbers of n and s substitutions on each branch, apparently, we confirmed that positive selection affected the evolution of angiosperm *PIN1* protein-coding sequences, but this effect was often masked by forces associated with purifying selection.

## Conclusion

PIN1 is an important auxin transporter and regulates multiple pathways involved in development. From algae to angiosperm the endogenous auxin is IAA for which transport is regulated by the highly conserved *PIN* families. Our analysis shows that angiosperm PIN1 orthologs contain highly conserved stretches of residues associated with the transmembrane and hydrophilic regions, which is consistent with the function of PIN1. Some angiosperm species contain two or more PIN1 homologs. PIN1 from Poaceae and Brassicaceae represent the modern clade because members of these families do not cluster with the AmtPIN1 sequence. We found 12 highly conserved motifs within PIN1 and a significant number of family-specific sites. This combination of family-specific sequence variations and conserved motifs, i.e., basic units, may have provided mechanisms for maintaining protein function as PIN1 of new species formed. One family-specific site within Motif 11 is functionally important, as it regulates PIN1 polarity. The amino acid at this site differs for monocot and dicot PIN1. There is very little evidence to suggest that PIN1 has different functions in monocots and dicots, although the phenotype associated with PIN1 overexpression is opposite in Arabidopsis and rice (Figure S5) [38], [39]. During the evolution of angiosperm *PIN1* protein-coding sequences purifying selection was the primary driver, but there are traces of positive selection associated with the formation of new orthologs. We verified this point by calculating the numbers of n and s substitutions for each branch of the phylogenetic tree containing 24 typical *PIN1* protein-coding sequences from the modern clade (Poaceae and Brassicaceae), Fabaceae, and the ancient clade (*VvPIN1a*, *CpPIN1*, and *AmtPIN1*).

To date, research concerning PIN1 has primarily focused on its function during development, but very few studies have addressed the origin of PIN1 sequences and their evolutionary trajectories. This paper not only shows the evolutionary processes of angiosperm *PIN1*, also finds a evolutionary way of the conservative function gene. At the same time, there are some questions to answer. For example, what are the functional consequences when an amino acid of one gene which needs to maintain the function is mutated during evolution? Finally, we did not analyze *PIN1* protein-coding sequences of gymnosperm because there was not enough sequence data available.

## Supporting Information

Figure S1 Average non-synonymous (*dN*) and synonymous (*dS*) distances associated with sequences from different families. “*” the Rutaceae genes, CcPIN1 and CsPIN1, had only a single nucleotide substitution, which led to a synonymous site. Thus, for Rutaceae, *dS* = 0.002 and there is no value associated with *dN*.
(TIF)

Figure S2 Maximum-likelihood phylogenetic tree for 38 typical angiosperm PIN1 sequences. The ML tree was constructed based on the protein sequences of angiosperm PIN1 using MEGA5.2 with 1000 bootstrap replications and Jones-Taylor-Thornton (JTT) + Gamma Distributed model (Discrete Gamma Categories = 5).
(TIF)

Figure S3 Motif distributions associated with 38 typical angiosperm PIN1 sequences.
(TIF)

Figure S4 Sequence logos of motifs identified in 38 typical angiosperm PIN1 sequences. Black arrows means non-conserved sites and the bright blue arrow means the important “Ser” site which decides the function and location of PIN1 in *Arabidopsis thaliana*. In Motif 4 and 9, three violet lines mark the conserved domain in PIN family gene and three violet squares mark the important “Ser” site which decides the function of PIN family gene.
(TIF)

Figure S5 PIN1 overexpression has different effects in rice (A) and *Arabidopsis* (B).
(TIF)

Table S1 The List of *PIN1* orthologs in this article.
(DOCX)

Table S2 Branch model test for each family PIN1 sequences.
(DOC)
