# Supplementary figures and images for: Phylogeny and Molecular Evolution Analysis of PIN-FORMED 1 in Angiosperm
Source: PLoS One. 2014 Feb 28;9(2):e89289. doi: 10.1371/journal.pone.0089289 (PMC3938449; doi:10.1371/journal.pone.0089289)

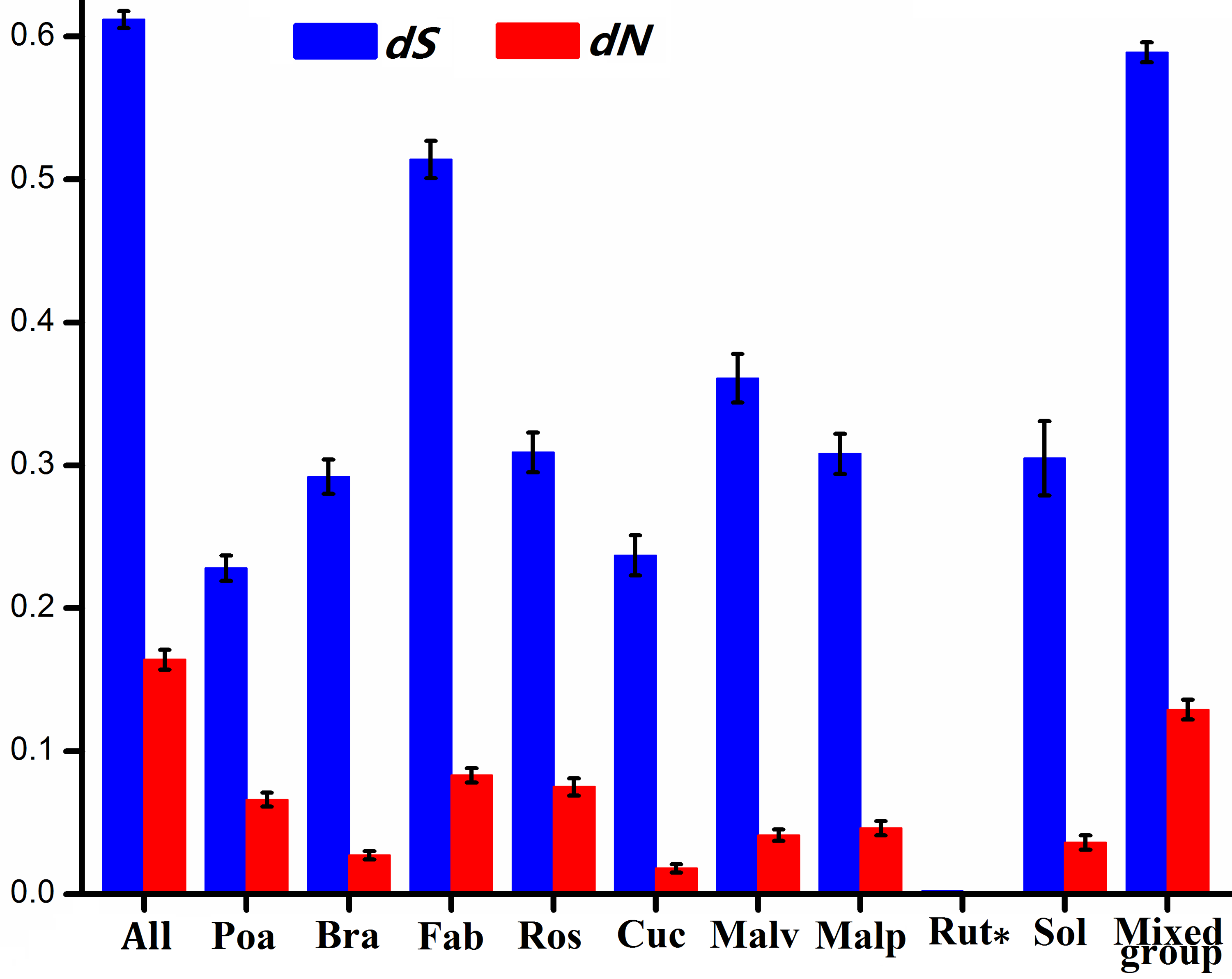

Supplement: Figure S1 — Average non-synonymous (dN) and synonymous (dS) distances associated with sequences from different families. “*” the Rutaceae genes, CcPIN1 and CsPIN1, had only a single nucleotide substitution, which led to a synonymous site. Thus, for Rutaceae, dS = 0.002 and there is no value associated with dN. (TIF) [file pone.0089289.s001.tif]

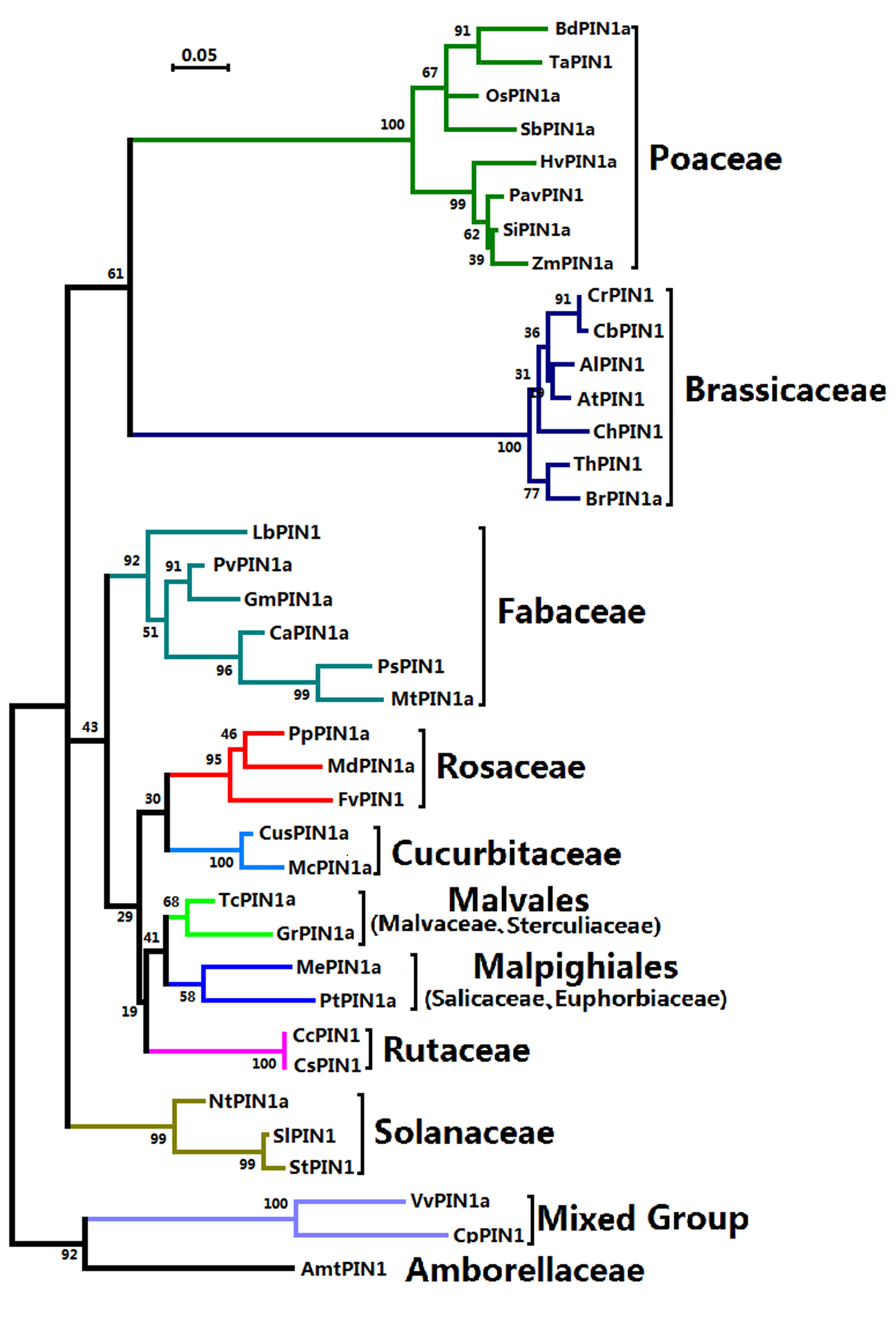

Supplement: Figure S2 — Maximum-likelihood phylogenetic tree for 38 typical angiosperm PIN1 sequences. The ML tree was constructed based on the protein sequences of angiosperm PIN1 using MEGA5.2 with 1000 bootstrap replications and Jones-Taylor-Thornton (JTT) + Gamma Distributed model (Discrete Gamma Categories = 5). (TIF) [file pone.0089289.s002.tif]

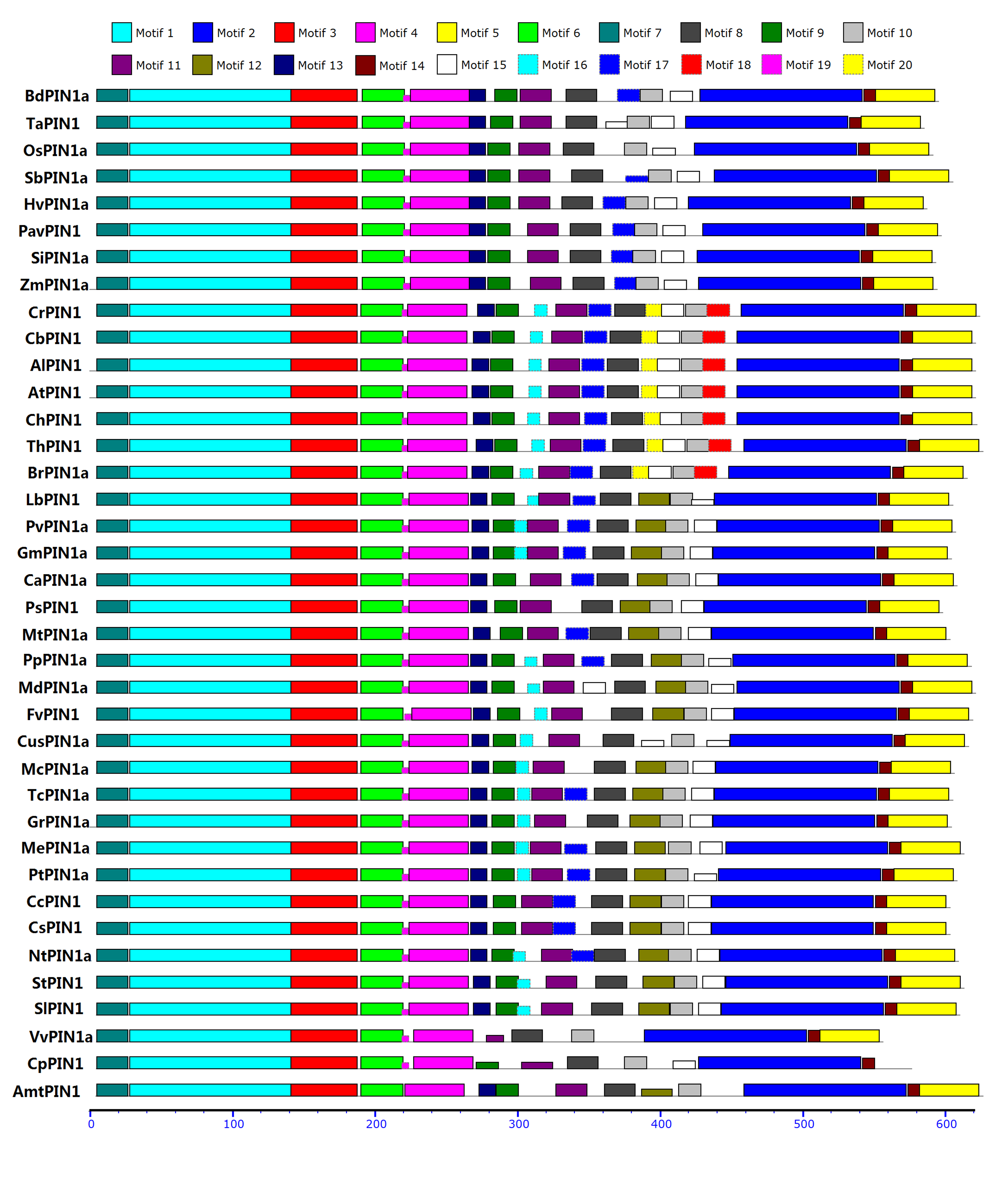

Supplement: Figure S3 — Motif distributions associated with 38 typical angiosperm PIN1 sequences. (TIF) [file pone.0089289.s003.tif]

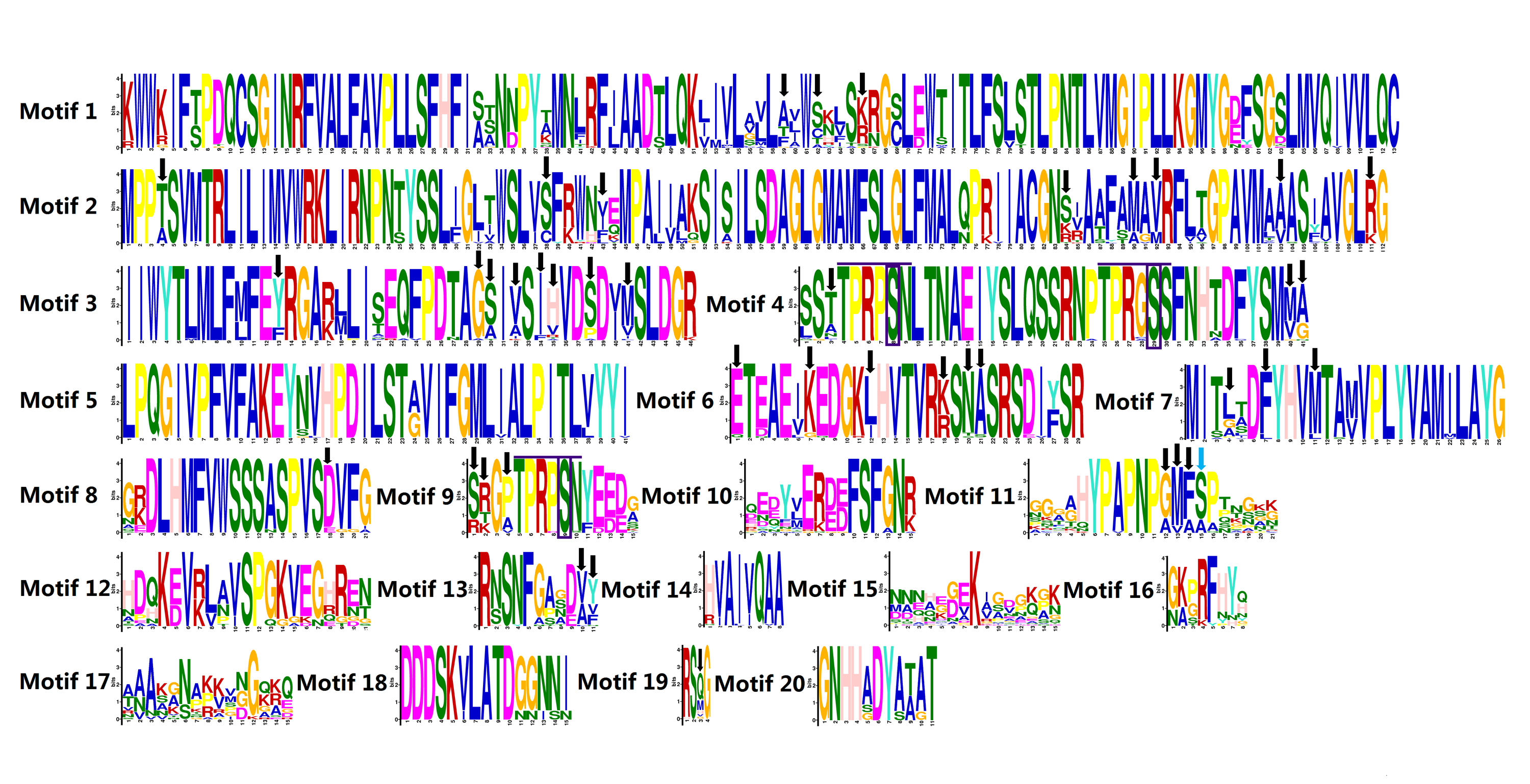

Supplement: Figure S4 — Sequence logos of motifs identified in 38 typical angiosperm PIN1 sequences. Black arrows means non-conserved sites and the bright blue arrow means the important “Ser” site which decides the function and location of PIN1 in Arabidopsis thaliana. In Motif 4 and 9, three violet lines mark the conserved domain in PIN family gene and three violet squares mark the important “Ser” site which decides the function of PIN family gene. (TIF) [file pone.0089289.s004.tif]

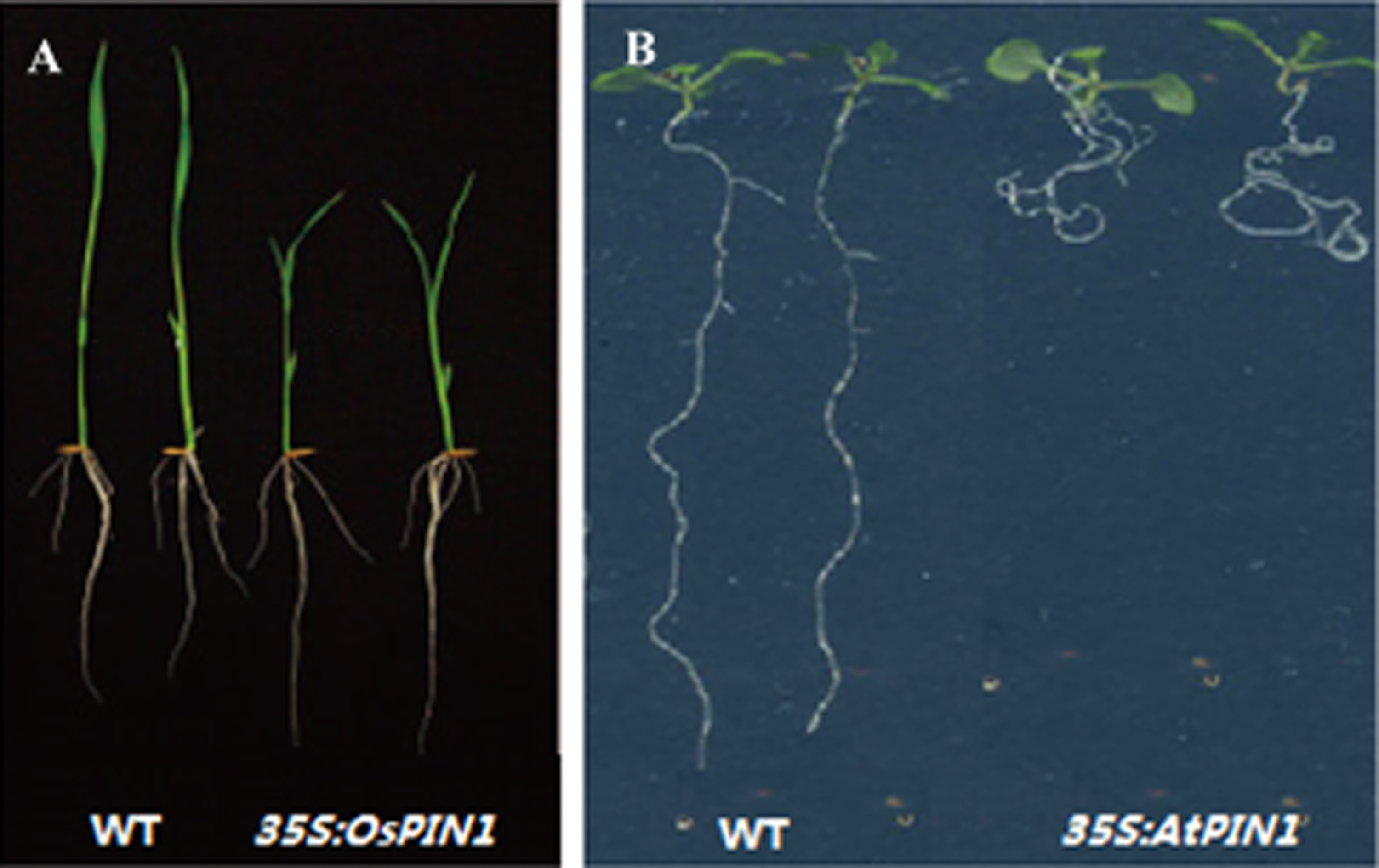

Supplement: Figure S5 — PIN1 overexpression has different effects in rice (A) and Arabidopsis (B). (TIF) [file pone.0089289.s005.tif]
