# Supplementary material for: Phylogeny and Molecular Evolution Analysis of PIN-FORMED 1 in Angiosperm
Source: PLoS One. 2014 Feb 28;9(2):e89289. doi: 10.1371/journal.pone.0089289 (PMC3938449; doi:10.1371/journal.pone.0089289)
Supplement: Table S1 — The List of PIN1 orthologs in this article. (DOCX) [file pone.0089289.s006.docx]

**Table S1.** The List of PIN1 genes in this article.

| **No.** | **Gene Name** | **Protein NCBI ID** | **CDS NCBI ID** | **Species** | **Family**  **（Order）** |
| --- | --- | --- | --- | --- | --- |
| **1** | **BdPIN1a** | **XP_003564038.1** | **XM_003563990.1** | ***Brachypodium distachyon*** | **Poaceae** |
| **2** | **BdPIN1b** | **XP_003570666.1** | **XM_003570618.1** |  |  |
| **3** | **HvPIN1a** | **BAJ97950.1** | **AK366747.1** | ***Hordeum vulgare*** |  |
| **4** | **OsPIN1a** | **NP_001057227.1** | **NM_001063762.1** | ***Oryza sativa*** |  |
| **5** | **OsPIN1b** | **NP_001048095.1** | **NM_001054630.1** |  |  |
| **6** | **PavPIN1** | **―** | **―** | ***Panicum virgatum*** |  |
| **7** | **SbPIN1a** | **XP_002436761.1** | **XM_002436716.1** | ***Sorghum bicolor*** |  |
| **8** | **SbPIN1b** | **―** | **―** |  |  |
| **9** | **SiPIN1a** | **XP_004953880.1** | **XM_004953823.1** | ***Setaria italica*** |  |
| **10** | **SiPIN1b** | **XP_004965062.1** | **XM_004965005.1** |  |  |
| **11** | **TaPIN1** | **AAS19858.1** | **AY496058.1** | ***Triticum aestivum*** |  |
| **12** | **ZmPIN1a** | **ABH09243.1** | **DQ836240.1** | ***Zea mays*** |  |
| **13** | **ZmPIN1b** | **ACB55418.1** | **EU570251.1** |  |  |
| **14** | **ZmPIN1c** | **ABH09242.1** | **DQ836239.1** |  |  |
| **15** | **AlPIN1** | **XP_002887490.1** | **XM_002887444.1** | ***Arabidopsis lyrata*** | **Brassicaceae** |
| **16** | **AtPIN1** | **NP_177500.1** | **NM_106017.3** | ***Arabidopsis thaliana*** |  |
| **17** | **BrPIN1a** | **―** | **―** | ***Brassica rapa*** |  |
| **18** | **BrPIN1b** | **―** | **―** |  |  |
| **19** | **CbPIN1** | **AEK01107.1** | **JN051352.1** | ***Capsella bursa-pastoris*** |  |
| **20** | **ChPIN1** | **ACH91863.1** | **FJ147293.1** | ***Cardamine hirsuta*** |  |
| **21** | **CrPIN1** | **EOA33778.1** | **KB870806.1** | ***Capsella rubella*** |  |
| **22** | **ThPIN1** | **―** | **―** | ***Thellungiella halophila*** |  |
| **23** | **CaPIN1a** | **XP_004512743.1** | **XM_004512686.1** | ***Cicer arietinum*** | **Fabaceae** |
| **24** | **CaPIN1b** | **XP_004503270.1** | **XM_004503213.1** |  |  |
| **25** | **GmPIN1a** | **AGJ95052.1** | **KC344366.1** | ***Glycine max*** |  |
| **26** | **GmPIN1b** | **AGJ95058.1** | **KC344372.1** |  |  |
| **27** | **GmPIN1c** | **NP_001267515.1** | **NM_001280586.1** |  |  |
| **28** | **GmPIN1d** | **NP_001267492.1** | **NM_001280563.1** |  |  |
| **29** | **LaPIN1** | **CAJ84441.1** | **AM235388.2** | ***Lupinus albus*** |  |
| **30** | **MtPIN1a** | **XP_003619829.1** | **XM_003619781.1** | ***Medicago truncatula*** |  |
| **31** | **MtPIN1b** | **XP_003631123.1** | **XM_003631075.1** |  |  |
| **32** | **PsPIN1** | **AAO38045.1** | **AY222857.1** | ***Pisum sativum*** |  |
| **33** | **PvPIN1a** | **―** | **―** | ***Phaseolus vulgaris*** |  |
| **34** | **PvPIN1b** | **―** | **―** |  |  |
| **35** | **FvPIN1** | **Fragaria vesca** | **XM_004299482.1** | ***Fragaria vesca*** | **Rosaceae** |
| **36** | **MdPIN1a** | **―** | **―** | ***Malus domestica*** |  |
| **37** | **MdPIN1b** | **―** | **―** |  |  |
| **38** | **MdPIN1c** | **―** | **―** |  |  |
| **39** | **MdPIN1d** | **―** | **―** |  |  |
| **40** | **PpPIN1a** | **EMJ11481.1** | **KB639086.1** | ***Prunus persica*** |  |
| **41** | **PpPIN1b** | **EMJ05411.1** | **KB639097.1** |  |  |
| **42** | **CusPIN1a** | **XP_004137502.1** | **XM_004137454.1** | ***Cucumis sativus*** | **Cucurbitaceae** |
| **43** | **CusPIN1b** | **XP_004144328.1** | **XM_004144280.1** |  |  |
| **44** | **McPIN1a** | **AAQ14257.1** | **AF247004.1** | ***Momordica charantia*** |  |
| **45** | **McPIN1b** | **AAQ14256.1** | **AF246995.1** |  |  |
| **46** | **GrPIN1a** | **―** | **―** | ***Gossypium raimondii*** | **Malvaceae、Sterculiaceae (Malvales)** |
| **47** | **GrPIN1b** | **―** | **―** |  |  |
| **48** | **GrPIN1c** | **―** | **―** |  |  |
| **49** | **TcPIN1a** | **EOY21347.1** | **―** | ***Theobroma cacao*** |  |
| **50** | **TcPIN1b** | **EOY08798.1** | **―** |  |  |
| **51** | **MePIN1a** | **―** | **―** | ***Manihot esculenta*** | **Salicaceae、Euphorbiaceae (Malpighiales)** |
| **52** | **MePIN1b** | **―** | **―** |  |  |
| **53** | **MePIN1c** | **―** | **―** |  |  |
| **54** | **PtPIN1a** | **XP_002317874.1** | **XM_002317838.1** | ***Populus trichocarpa*** |  |
| **55** | **PtPIN1b** | **XP_002322104.1** | **XM_002322068.1** |  |  |
| **56** | **PtPIN1c** | **XP_002307966.1** | **XM_002307930.1** |  |  |
| **57** | **PtPIN1d** | **XP_002322614.1** | **XM_002322578.1** |  |  |
| **58** | **CcPIN1** | **―** | **―** | ***Citrus clementina*** | **Rutaceae** |
| **59** | **CsPIN1** | **―** | **―** | ***Citrus sinensis*** |  |
| **60** | **NtPIN1a** | **AGG79239** | **KC347302.1** | ***Nicotiana tabacum*** | **Solanaceae** |
| **61** | **NtPIN1b** | **AGG79244.1** | **KC460399.1** |  |  |
| **62** | **SlPIN1** | **NP_001234163.1** | **NM_001247234.1** | ***Solanum lycopersicum*** |  |
| **63** | **StPIN1** | **―** | **―** | ***Solanum tuberosum*** |  |
| **64** | **VvPIN1a** | **XP_002282693.1** | **XM_002282657.1** | ***Vitis vinifera*** | **Vitaceae** |
| **65** | **VvPIN1b** | **CBI32786.3** | **FN596008.1** |  |  |
| **66** | **CpPIN1** | **―** | **―** | ***Carica papaya*** | **Caricaceae** |
| **67** | **AmtPIN1** | **―** | **―** | ***Amborella trichopoda*** | **Amborellaceae** |

**“—” means the sequences were searched in the phytozome website (http://www.phytozome.org).**
