# Supplementary material for: Phylogeny and Molecular Evolution Analysis of PIN-FORMED 1 in Angiosperm
Source: PLoS One. 2014 Feb 28;9(2):e89289. doi: 10.1371/journal.pone.0089289 (PMC3938449; doi:10.1371/journal.pone.0089289)
Supplement: Table S2 — Branch model test for each family PIN1 sequences. (DOC) [file pone.0089289.s007.doc]

**Table S2.** Branch model test for each family PIN1 genes.

| **Subfamily** | ***dN*/*dS***  **one-ratio** | **lnL** | | **2Δl** | **P value** |
| --- | --- | --- | --- | --- | --- |
|  |  | **one-ratio** | **free-ratio** |  |  |
| Poaceae | 0.037 | -6749.914 | -6707.020 | 85.788 | 0.000 |
| Brassicaceae | 0.063 | -5446.953 | -5435.138 | 23.630 | 0.000 |
| Fabaceae | 0.084 | -7248.172 | -7182.545 | 131.254 | 0.000 |
| Rosaceae | 0.084 | -4698.308 | -4669.429 | 57.742 | 0.000 |
| Cucurbitaceae | 0.042 | -2964.887 | -2957.035 | 15.704 | 0.000 |
| Malvales | 0.081 | -3730.496 | -3724.541 | 11.910 | 0.360 |
| Malpighiales | 0.085 | -4230.855 | -4226.724 | 8.262 | 0.004 |
| Solanaceae | 0.083 | -3286.387 | -3285.514 | 1.746 | 0.186 |
| Mixed group | 0.055 | - 8962.260 | -9213.284 | 502.048 | 0.000 |

The single *dN*/*dS* value (average over all sites) for each family (order) PIN1 genes was obtained by M0 model (one-ratio model);

lnL: the log-likelihood difference between the two models; 2Δl: twice the log-likelihood difference between the two models；

In Poaceae, Brassicaceae ,Fabaceae, Rosaceae, Cucurbitaceae, Malpighiales, and Mixed group, the free-ratio model is favored (p<0.05), suggesting that there are variable selective pressures.
